# A Protocol for Dual Calcium-Voltage Optical Mapping in Murine Sinoatrial Preparation With Optogenetic Pacing

**DOI:** 10.3389/fphys.2019.00954

**Published:** 2019-08-06

**Authors:** Ruirui Dong, Razik Mu-u-min, Alastair J. M. Reith, Christopher O’Shea, Shicheng He, Kaizhong Duan, Kun Kou, Alexander Grassam-Rowe, Xiaoqiu Tan, Davor Pavlovic, Xianhong Ou, Ming Lei

**Affiliations:** ^1^Key Laboratory of Medical Electrophysiology of Ministry of Education and Medical Electrophysiological Key Laboratory of Sichuan Province, Institute of Cardiovascular Research, Southwest Medical University, Luzhou, China; ^2^Department of Pharmacology, University of Oxford, Oxford, United Kingdom; ^3^Institute for Cardiovascular Sciences, University of Birmingham, Birmingham, United Kingdom

**Keywords:** sinoatrial preparation, optogenetic pacing, optical mapping, murine heart, Ca^2+^ transient

## Abstract

Among the animal models for studying the molecular basis of atrial and sinoatrial node (SAN) biology and disease, the mouse is a widely used species due to its feasibility for genetic modifications in genes encoding ion channels or calcium handling and signaling proteins in the heart. It is therefore highly valuable to develop robust methodologies for studying SAN and atrial electrophysiological function in this species. Here, we describe a protocol for performing dual calcium-voltage optical mapping on mouse sinoatrial preparation (SAP), in combination with an optogenetic approach, for studying SAP membrane potential, intracellular Ca^2+^ transients, and pacemaker activity. The protocol includes the details for preparing the intact SAP, robust tissue dual-dye loading, light-programmed pacing, and high-resolution optical mapping. Our protocol provides an example of use of the combination of optogenetic and optical mapping techniques for investigating SAP membrane potential and intracellular Ca^2+^ transients and pacemaker activity with high temporal and spatial resolution in specific cardiac tissues. Thus, our protocol provides a useful tool for studying SAP physiology and pathophysiology in mice.

## Introduction

The heartbeat begins in the sinoatrial node (SAN) arising in a subset of nodal cells that display a spontaneous diastolic depolarization (Bleeker et al., 1980). Unique to such automaticity of the SAN, pacemaker cells possess “membrane clock” contributions by several membrane currents including the funny current, I_f_, carried by hyperpolarization-activated cyclic nucleotide-gated (HCN) channels. I_f_ is initiated specifically at the initial phase of the diastolic depolarization, following the deactivation of outward delayed rectifier K^+^ current, and triggering the activation of inward currents. Inward currents include Na^+^-dependent background current; the T- and L-type Ca^2+^ currents, I_CaT_ and I_CaL_; and possibly, sustained inward current, I_st_ (Lei et al., 2018). However, more recently, intracellular signaling involving the sarcoplasmic reticulum (SR) Ca^2+^ stores and the cellular cAMP levels and consequent phosphorylation of their signaling proteins have also been implicated in a “Ca^2+^ clock” contributing to diastolic depolarization (Lei et al., 2018). Whereby, spontaneous RyR2-mediated Ca^2+^ release may enhance electrogenic effects of the Na^+^-Ca^2+^ exchanger in late diastolic depolarization (Lei et al., 2018). These SR calcium release events may be triggered by calcium-induced calcium release following the activation of Ca_V1.3_ calcium channels (Torrente et al., 2016). The relative importance of both the “membrane clock” and “Ca^2+^ clock” in pacemaker function remains controversial.

On the other hand, sinus node dysfunction (SND) associated with abnormal impulse formation or propagation in the SAN affects ≈1 in 600 cardiac patients aged >65 years and is responsible for ≈50% of the 1 million permanent pacemaker implants per year worldwide (de Marneffe et al., 1993). Although SND occurs most commonly in elderly patients in the absence of clinically apparent accompanying cardiac disease (de Marneffe et al., 1993), its pathogenesis is unclear. The development of novel pharmacological therapies to cure these patients relies on the thorough understanding of both normal physiology and pathophysiology of the SAN and atria.

Among the animal models for studying the basic molecular mechanisms of the SAN and atrial biology and disease, mice are widely used due to their feasibility for modifications in the expression of different genes that encode ion channels or calcium handling proteins. It is therefore highly valuable to develop robust methodologies and techniques for studying SAN and atrial electrophysiological properties.

Recent advances in the technique of optogenetics provide an unprecedented opportunity for defining specific cell-type function in a highly complex multicellular system. The technique has enabled control over the activity of one specific cell type while leaving others unaltered, with high temporal resolution and cellular precision. Since the first demonstrations of utility in mammalian neurons in 2005 (Boyden et al., 2005), optogenetics has spurred immense research activity in neuroscience and has extended to the cardiac field over the past few years (Entcheva, 2013; Nussinovitch and Gepstein, 2015; Boyle et al., 2018). We recently deployed a cell-type specific optogenetic approach to study the properties of Pnmt^+^ cell-derived cardiomyocytes in the murine heart (Wang et al., 2017). Additionally, optical mapping using fluorescent dyes provides exciting opportunities for high spatio-temporal study of cellular electrophysiological events, including Ca^2+^ dynamics of the heart (Wang et al., 2017; Wen et al., 2018). The combination of these two technologies therefore allows development of unprecedented platforms for studying physiological events (e.g., voltage and Ca^2+^ signals) in a selective manner at high spatio-temporal resolution (O’Shea et al., 2019b).

In this manuscript, we describe a detailed protocol for the use of optogenetic and optical mapping techniques for investigating membrane potential and intracellular Ca^2+^ transients and pacemaker activity with high temporal and spatial resolution in sinoatrial preparations (SAPs). The protocol can be a powerful tool for studying SAP physiology and pathophysiology using the mouse as a model system.

## Methods and Materials

### Animals

Pnmt-cre mice used in this study are as described previously (Wang et al., 2017). Animals used were <6 months old. Pnmt-cre/ChR2 mice exhibit cell-type specific expression of ChR2 by crossing B6.Cg-*Gt (ROSA)26Sor^tm27.1(CAG-COP4H134R/tdTomato) Hze^*/J strain (Stock no. 012567, Jackson Labs) with a Cre transgenic strain under the control of a *Pnmt* promoter (Wang et al., 2017). Channelrhodopsin 2 (ChR2) was specifically introduced into murine cells expressing the *Phenylethanolamine n-methyltransferase* (*Pnmt*) gene, which encodes for the enzyme responsible for conversion of noradrenaline to adrenaline. The murine model led to the identification of a distinctive class of Pnmt-expressing neuroendocrine cells and their descendants (i.e., Pnmt^+^ cell-derived cardiomyocytes) within the heart (Wang et al., 2017).

All procedures have been approved by Institutional Animals Ethics Committees at Southwest Medical University, Luzhou, China or Department of Pharmacology at University of Oxford, UK and the national guidelines under which the institution operates. All mice used in this study were maintained in a pathogen-free facility at Southwest Medical University or University of Oxford. Mice were given *ad libitum* access to food and water. The authors confirm that they have taken all steps to minimize the animals’ pain and suffering.

### Materials and Equipment

Table 1 shows the details of materials and buffers.

**Table 1:** Details of materials and buffers.

Chemical and catalogue reference	Supplier
NaCl (SLBS2340V)	Sigma-Aldrich, St Louis, MO, USA
NaHCO_3_ (SLBX3605)	Sigma-Aldrich
NaH_2_PO_4_ (BCBW9042)	Sigma-Aldrich
KCl (SLBS5003)	Sigma-Aldrich
MgCl_2_ (BCBS6841V)	Sigma-Aldrich
CaCl_2_ (SLBK1794V)	Sigma-Aldrich
Glucose (SLBT4811V)	Sigma-Aldrich
HEPES (W1122DO10)	Sangon biological Shanghai, China
2,3-butanedione monoxime (BDM) (29297)	Sigma-Aldrich
Blebbistatin (SLBV5564)	Tocris Bioscience Minneapolis, MN, USA
Voltage-sensitive dye RH237 (1971387)	Thermo Fisher Scientific, Waltham, MA, USA
Calcium indicator Rhod-2 AM (1890519)	Invitrogen, Carlsbad, CA, USA
Dimethyl sulfoxide (DMSO) (RNBT7442)	Sigma-Aldrich
Heparin Sodium (H51021209)	Chengdu Haitong Pharmaceutical Co. Ltd. Chengdu, China
Avertin (2,2,2-tribromoethanol)	Sigma-Aldrich Poole, Dorset, UK
Pluronic F127 (1899021)	Invitrogen, Carlsbad, CA, USA

**Figure 1:**
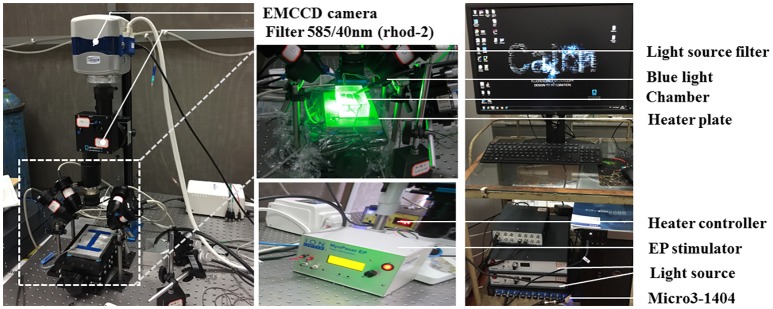
The optical imaging system. Two green (a peak wavelength of 530 nm) and two red (a peak wavelength of 627 nm) LED lights (Cairn Research, Faversham, Kent, UK) were placed around the chamber so as to uniformly illuminate the SAP. The green LEDs were fitted with excitation bandpass filters (S555/25X, Chroma Technology GmbH, Germany), and the red LEDs were fitted with excitation bandpass filters (HQ640/50, Chroma Technology GmbH, Germany) to efficiently select the best excitation wavelength range for Rhod-2 AM and Di-4-ANBDQPQ, respectively. A multi-bandpass emission filter (ET595/50 + 700LP, Chroma Technology GmbH, Germany) was placed in front of the camera lens to avoid bleed-through of the excitation light and to selectively let the fluorescence emission pass through. A multi-stream light switcher (Cairn Research, Faversham, Kent, UK) was set up to automatically switch between the red and green LED lights in accordance with the frame rate of the camera. Signal recording using this setup is in such a way that one frame data recorded will have only the red light turned on (thereby recording voltage signal alone) and the next frame data recorded will have only the green light turned on (thereby recording CaT signal alone), and the process is repeated until the desired number of frames are recorded.

Figure 1 shows the optical imaging system for light stimulation of ChR2 light-sensitive channels and optical recording.

The tissues were paced through the activation of ChR2 light-sensitive channels. This was achieved by the delivery of 470 nm blue light pulses (13–14 ms pulse width) generated by OptoFlash (Cairn Research, Faversham, UK). Pulses were triggered by a 1,401 digitizer and Spike 2 software (Cambridge Electronic Design). Approximate blue light intensity was measured with a 818-ST2 Wand Detector connected to a 843-R power meter (both Newport Corporation, CA, USA), and we estimate an average irradiance in our experiments of 0.1–0.3 mW/mm^2^ based on an approximate distance of 1–2 cm between Sylgard and liquid light guide (Oriel instruments Model No. 77525).

### Buffer Compositions

#### Physiological Salt Solution

Freshly prepared physiological salt solution (PSS) containing in mM: NaCl 119, NaHCO_3_ 25, NaH_2_PO_4_ 1.0, KCl 4.7, MgCl_2_ 1.2, CaCl_2_ 1.35, and glucose 10; equilibrated with 95% O_2_/5% CO_2_ at 37°C, pH = 7.35.

#### Dye Loading Solution

Calcium indicator Rhod-2 AM (Invitrogen, Carlsbad, CA, USA) was dissolved in dimethyl sulfoxide (DMSO, Sigma-Aldrich, St. Louis, MO, USA) to prepare a stock concentration of 10 mg/ml (8.9 mM). The voltage indicator stock was prepared by dissolving Di-4-ANBDQPQ (University of Connecticut Health Center) in ethanol to obtain a stock concentration of 29 mM. To avoid repeated freezing and thawing, both stocks were stored in 50 μl aliquots at −20°C. For working concentrations of Rhod-2 AM dye loading solution, 15 μl Rhod-2 AM stock solution was mixed with 15 μl Pluronic F-127 (20% solution in DMSO, Invitrogen, Carlsbad, CA, USA) and then dissolved in 15 ml PSS (10 μM final concentration). Similarly, 5.2 μl of Di-4-ANBDQPQ stock was mixed with 15 μl Pluronic F-127 and then dissolved in 15 ml PSS to form a working concentration of Di-4-ANBDQPQ dye loading solution (10 μM final concentration). The working dye loading solutions were prepared on the day of the experiment.

#### Blebbistatin Physiological Salt Solution

Excitation-contraction uncoupler blebbistatin (Invitrogen, Carlsbad, CA, USA) was dissolved in in DMSO (Sigma-Aldrich, St. Louis, MO, USA) to make up 10 mM stock solution. To avoid repeated freezing and thawing, aliquots of 15 μl were stored at −20°C. A 10 μl of 10 mM stock blebbistatin solution was added to 15 ml PSS to make blebbistatin PSS solution (10 μM final concentration).

#### Preparation and Equipment

1. Dissection set-up includes stereomicroscope, dissection chamber filled with Sylgard gel, oxygenation device, LED light source, and surgical instruments.
2. The optical mapping system was set up as detailed below:

A custom-designed system equipped with an EMCCD camera (Evolve 128, Photometrics, Tucson, AZ, USA) was used (see Figure 1). Two green (to excite Ca^2+^ sensitive dye Rhod-2 AM; a peak wavelength of 530 nm) and two red (to excite the voltage sensitive dye Di-4-ANBDQPQ; a peak wavelength of 627 nm) LED lights (Cairn Research, Faversham, UK) were placed around the imaging chamber so as to uniformly illuminate the SAP. The green LEDs (peak wavelength = 530 nm) were fitted with excitation bandpass filters (S555/25X, Chroma Technology GmbH, Germany), and the red LEDs (peak wavelength = 627 nm) were fitted with excitation bandpass filters (HQ640/50, Chroma Technology GmbH, Germany) to efficiently select optimal excitation wavelength range for Rhod-2 AM and Di-4-ANBDQPQ, respectively. A multi-bandpass emission filter (ET595/50 + 700LP, Chroma Technology GmbH, Germany) was placed in front of the camera lens to avoid bleed-through of the excitation light and to selectively let the fluorescence emission to pass through. CaT (calcium transient) and transmembrane potential (*V*_m_) measurements were taken at high resolution (128 × 128 pixels; pixel size 66 × 66 μm) at a rate of 520 frames/s.

## Protocol

### Harvest the Mouse Heart and Prepare the Sinoatrial Preparation (30 min)

**Figure 2:**
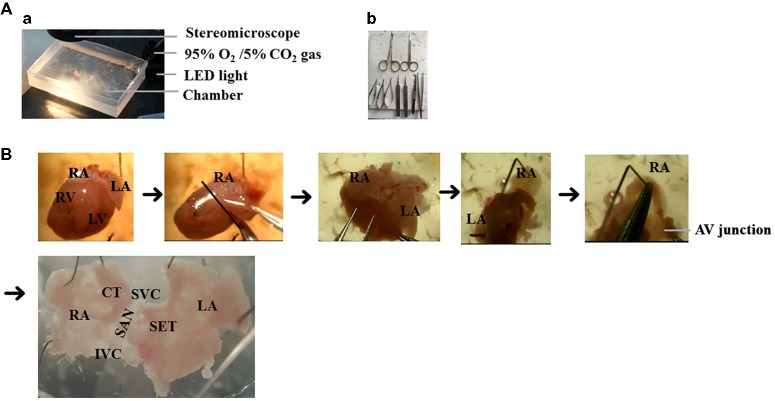
The isolation of mouse SAP. **(A)** The setup of dissection. **(a)** The dissection was performed under the stereomicroscope and oxygenated PSS solution. **(b)** Surgical instruments. **(B)** The preparation of SAP. RA, right atrium; LA, left atrium; RV, right ventricle; LV, left ventricle; SAN, sinoatrial node; AV junction, atrioventricular junction, SET = septum, SVC = superior vena cava, IVC = inferior vena cava, CT = crista terminalis.

The steps for preparation of the SAP are outlined in Figure 2 and as described previously (Liu et al., 2007; Hao et al., 2011; Sharpe et al., 2016). The details are as follows:

1. Non-recovery terminal general anesthesia was induced by injection of an overdose of 1.2% Avertin solution (0.5–0.8 ml i.p., 2,2,2-tribromoethanol, Sigma-Aldrich, Poole, Dorset, UK), followed by intraperitoneal injection with heparin (200 units).
2. Following sacrifice *via* cervical dislocation, the heart was rapidly excised and placed in warmed (37°C) and oxygenated (95% O_2_ + 5% CO_2_ gas) PSS solution in a dissection chamber.
3. The heart was gently pressed with the tips of fingers to push the blood out of heart. Fresh oxygenated PSS was then added for further dissection.
4. SAP containing two atria and the SAN region was dissected after clearing off the lung, thymus, ventricles, and connective tissues (Figure 2B).
5. The right atrium was opened from atrioventricular (AV) junction to expose the intercaval region containing the SAN, following which left atrium was opened to expose the left atrial endocardium. As the pacemaker cells are widely distributed throughout the entire region located between the superior (SVC) and inferior vena cava (IVC) and between the crista terminalis and intra-atrial septum (Glukhov et al., 2010), the SAP contains the SAN and the two atria intact.
6. The SAP was pinned onto a small rectangular piece of Sylgard gel by using fine insect pins. All dissection steps were performed in a dissection chamber with oxygenated PSS solution under a stereomicroscope.

### Dual Dye Loading of the Sinoatrial Preparation (25 min)

7. A 50 ml falcon tube was filled with 15 ml of the Rhod-2 AM dye loading solution.
8. The Sylgard gel (with SAP attached) was then dropped into the falcon tube.
9. The SAP-containing falcon tube was placed in a 36°C water bath. The solution was oxygenated constantly by gently bubbling with 95% O_2_/5% CO_2_ (Note: heavy bubbling can lead to the formation of foam due to the presence of Pluronic F-127 in the solution. This can lead to the suboptimal dye loading). The SAP was incubated in the dye loading solution for 15 min.
10. Dual dye loading of the SAP was performed by incubating the SAP in Rhod-2 AM dye loading solution as detailed above (points 7–9), following which the Sylgard gel with SAP attached was transferred into a new 50 ml falcon tube containing 10 μM Di-4-ANBDQPQ dye loading solution. Incubation with Di-4-ANBDQPQ was performed for 10 min at 36°C and constantly bubbled with 95% O_2_/5% CO_2._

### Optical Mapping (30 min)

11. The imaging chamber was filled with 15 ml of blebbistatin PSS solution and was placed directly under the camera. The recording chamber was heated up to 36°C by a heating plate.
12. The Sylgard (with SAP attached) was removed from the falcon tube and pinned down in the imaging chamber. The sample was placed between the two field-stimulation electrodes. (Note: Removing the SAP from the Sylgard and pinning down directly in the chamber cause further damage to the SAP. Therefore, pinning down the Sylgard with the intact tissue is recommended). The blebbistatin PSS solution was bubbled in the imaging chamber with 95% O_2_/5% CO_2_ gas.
13. Imaging was performed after cessation of motion by blebbistatin. Imaging used the EMCCD camera controlled by the acquisition software Metamorph (Molecular Devices). During image recordings, the excitation lights were turned on, and bubbling was temporarily stopped (to avoid distortion of the signals due to bubbles and motion of the solution). Since Pnmt^+^ cell-derived cardiomyocytes (PdCMs) express channelrhodopsin-2 (ChR2), they can be excited by exposure to 470 nm blue light. The stimulation protocol was then run, and baseline calcium transient or action potential recordings were taken. Recordings were taken again after stimulating the left atrium using blue light (pacing intervals: 150–160 ms, pulse duration: 13–14 ms, intensity: 1,992 mA). The frame rate of these recordings was 0.52 kHz.
14. For dual dye imaging, a multistream light switcher (Cairn Research, Faversham, Kent, UK) was set up to automatically switch between the red (627 nm) and green (530 nm) LED lights according to the frame rate of the camera. Signal recording using this setup was performed, so that one frame of the data was recorded under red light illumination (thereby recording *V*_m_ signal alone), and the next frame was recorded under green light illumination (thereby recording CaT signal alone) and so forth until the desired number of frames was recorded. This resulted in an interlaced signal containing alternate frames of CaT and *V*_m_ signals. The interlaced signal was separated into CaT and voltage signal using ImageJ and saved as separate files. Due to the signals being interposed, the frame rate recording was 0.26 kHz.

### Analysis

15. The saved image files were loaded, processed, and analyzed using the optical mapping analysis software ElectroMap (developed by Dr. Pavlovic’s group, https://github.com/CXO531/ElectroMap; O’Shea et al., 2019a).
16. Images were pre-processed by applying a 4 × 4 pixel Gaussian spatial filter (standard deviation = 1.5), a Top-Hat baseline correction (kernel size = 100 ms), and a third order Savitzky-Golay temporal filter. A window size of 40 ms before transient peak to 100 ms after transient peak was used. Action potential duration (APD) and CaT duration (CaD) were measured at the desired repolarization/decay percentage at each pixel across the tissue, as measured from time of maximum upstroke velocity (maximum dF/dt).
17. For assessment of local conduction velocities, activation maps were generated using time of depolarization midpoint. Conduction velocity across the SAP was then quantified using a multi-vector polynomial method (Bayly et al., 1998) with a local window size of 5 × 5 pixels.
18. To further assess conduction across the entire SAP, we used activation curve analysis, where the percentage of tissue activated was plotted as a function of time (O’Shea et al., 2019a).

### Representative Results

**Figure 3:**
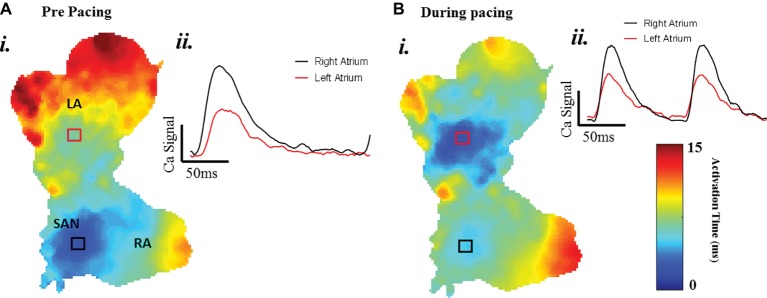
The change of atrial conduction in optogenetic mice during after blue light stimulation. Maps produced by mapping the midpoint time of the CaT rise. **(A)** Ca^2+^ transient activation map during intrinsic pacing, before optical pacing. **(B)** Ca^2+^ transient activation map during optical pacing with blue (470 nm) light pulses.

**Figure 4:**
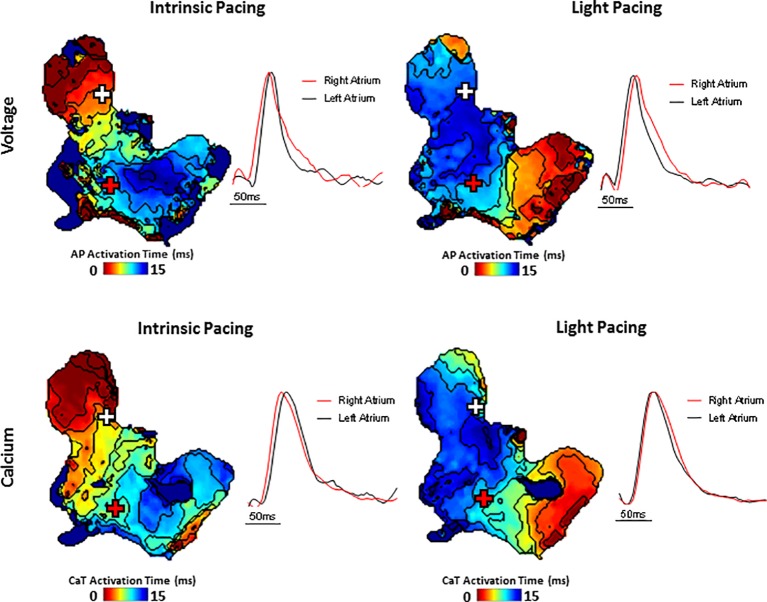
Simultaneous voltage (**top panels**) and Ca^2+^ transient (**bottom panels**) activation maps reconstructed from spontaneous sinus rhythmic (**left panels**) and light paced (**right panels**) SAP. Maps were produced by mapping the midpoint time of the voltage and CaT rise with examples of voltage and Ca^2+^ transient traces from indicated regions of the preparation (white and red cross).

A typical example of an SAP Ca^2+^ transient activation map reconstructed from spontaneous sinus rhythmic Ca^2+^ transients (by Rhod-2 AM) recorded with the optical mapping system is shown in Figure 3A for a SAP. The early activation point is located within the intercaval region near to where the SAN is located. However, as shown in Figure 3B, the calcium activation pattern and atrial conduction changed after stimulating with blue light (interval: 150 ms; duration: 13 ms; intensity: 1,992 mA; pulses per train: 50). The leading pacemaker site was shifted to the location of left atrium, suggesting that PdCMs were activated earlier than the SAN pacemaker cells. This is consistent with our previous report that PdCM cells predominantly localized to the left atrium and left ventricle, and the heart rate was controlled by stimulating PdCMs selectively with blue light (Wang et al., 2017). We hypothesize that PdCM cells overexpressing ChR2 may be a viable site for spatially-targeted optical pacing when sinus node pacemaker cells are dysfunctional. Further investigation is required. Figure 4 shows representative *V*_m_ and CaT maps and voltage and calcium traces from different regions of the SAP, illustrating the robustness of this preparation for murine research. We monitored the “rundown” of the *V*_m_ and CaT signals for up to 4 h – the average signal “rundown” is less than 25%. Our experiment usually finished within 3 h from the beginning of Langendorff perfusion of the heart. We again observe augmentation of the activation pattern induced by optical pacing, shown by both the voltage (Figure 4, upper panel) and calcium (Figure 4, lower panel) activation maps. Activation maps were produced by mapping the midpoint time of the AP/CaT rise.

**Figure 5:**
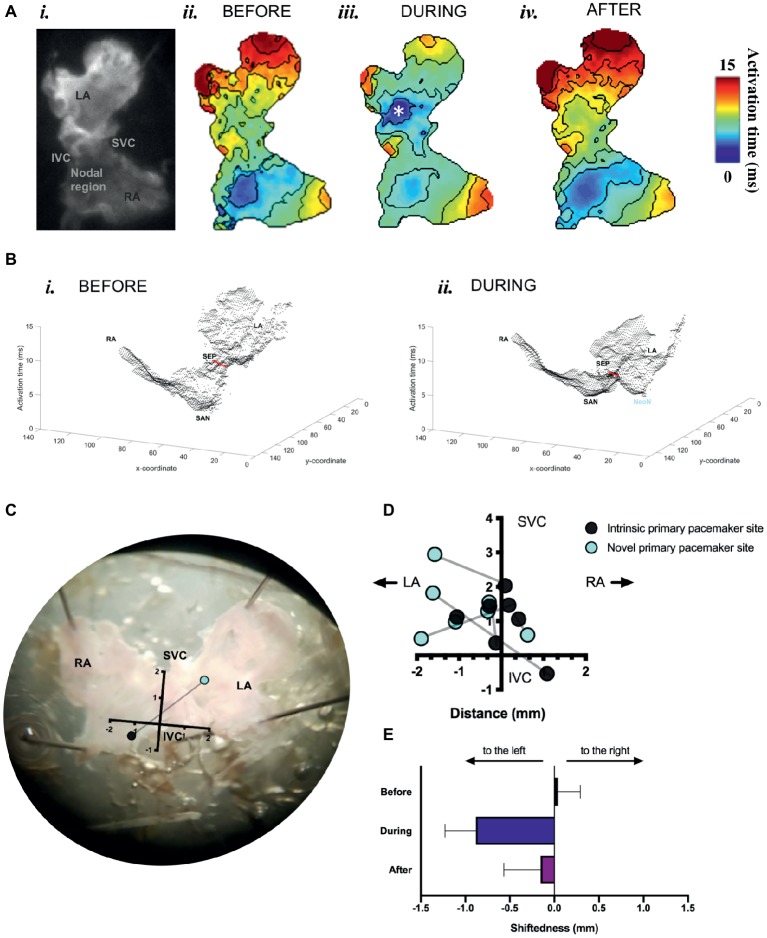
Mapping of activation in SAPs under sinus rhythm and under optogenetic pacing. **(A)** (*i*) Representative SAP—camera view. (*ii*) Activation map for this SAP before optogenetic pacing. (*iii*) Activation map during pacing (note formation of “neonode”, white asterisk). (*iv*) Activation map after pacing. Activation maps are calculated from Ca^2+^ transients. Fifty pacing spikes given, at 6.67 Hz. Contours drawn at 1/frame rate of the EMCCD. Scale bar, right. **(B)** 3-D graphical rendering of activation maps (*i*) before pacing, (*ii*) during pacing. SVC-IVC axis in red. Activation time on z-axis. **(C)** Representative SAP—microscope view. Pacemaker shift overlaid. SVC-IVC axis shown. Distances in mm. **(D)** SVC-IVC axis with relative pacemaker shifts plotted (*n* = 7). **(E)** Mean change in “shiftedness.” LA, left atrium; RA, right atrium; SAN, sinoatrial node; SVC, superior vena cava; IVC, inferior vena cava; SEP, septum; NeoN, neonode.

We found that optogenetic pacing of a SAP can shift the activation point to the left, creating a new leading pacemaker site or “neonode.” Activation patterns in the SAP were plotted using ElectroMap (O’Shea et al., 2019a), with the midpoint of the upstroke of the calcium transient used as a pixel-by-pixel readout of tissue activation. The earliest-activating pixel—discarding outliers—was designated the activation point for that tissue. The earliest activation point before pacing was always in the nodal region. Optogenetic pacing of PdCMs can move this activation point to the left (Figure 5), establishing a new leading pacemaker site toward the left atrium. This change in the location of the leading pacemaker is reversible upon cessation of pacing (Figures 5A,E). Left shift of the leading pacemaker occurred in four of seven SAPs (Figure 5D). One leftward deviation appears more upward relative to the SVC-IVC axis—this is due to contortion of the tissue. Although no leading pacemaker shift occurred in three SAPs, all seven SAPs were successfully paced by blue light: with a decrease in mean cycle length from 264.3 ± 42.7 ms before optogenetic pacing to 159.2 ± 1.4 ms during (*p* < 0.02). Mean cycle length after pacing was 281.9 ± 48.9 ms—not significantly different from before (*p* = 0.63). Therefore, we can always successfully and reversibly pace PdCMs using selective ChR2 overexpression. The variability in pacemaker shift observed (Figure 5D) is consistent with the variable distribution of murine PdCMs across both the SAN region and the left atrium (Ni et al., 2017; Wang et al., 2017). In summary, we report the possibility of using optogenetic pacing of PdCMs to establish novel left-shifted leading pacemaker sites in the murine heart.

**Figure 6:**
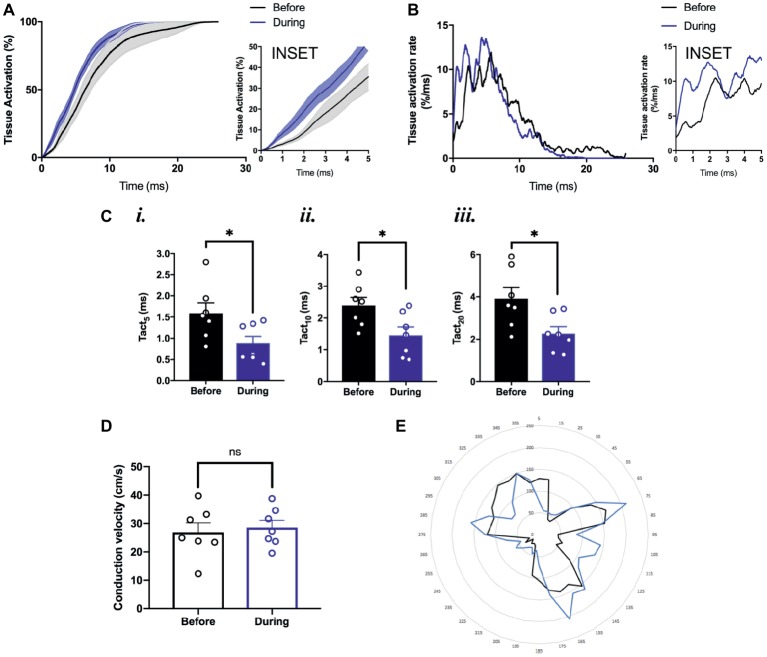
Conduction pattern in the tissue under sinus rhythm and during optogenetic pacing. **(A)** Grouped activation curve showing mean % tissue activated over the time elapsed during a Ca^2+^ transient. SEM denoted by shading. Inset: zoomed section of first 5 ms. **(B)** Rate of change of % tissue activation plotted against time. Inset as above. **(C)** (*i*) Time to 5% tissue activation. (*ii*) Time to 10% tissue activation. (*iii*) Time to 20% tissue activation. **(D)** Mean magnitude of local conduction vectors. **(A–D)** *n* = 7 throughout. **(E)** Representative radar plot, showing local conduction vector directionalities. Angles binned into groups of 10°—so 0° ≤ *x* < 10° becomes 5°, 10° ≤ *x* <°20° becomes 15°, and so on. Concentric circles represent increasing frequency of vectors at that angle.

To study the effects of optogenetic pacing on overall conduction, we utilized activation curve analysis where the percentage of tissue activated is plotted as a function of time (O’Shea et al., 2019a). Optogenetic pacing of PdCMs augments conduction in the SAP—producing a left shift of the activation curve (Figure 6A). This is most noticeable in the first few milliseconds of tissue activation (Figure 6A, inset). During optogenetic pacing, the rate of tissue activation is initially very rapid (Figure 6B, inset), otherwise, there are no major alterations to tissue activation rate (Figure 6B). Optogenetic pacing reduces the time to 5% tissue activation from 1.59 ± 0.24 to 0.88 ± 0.17 ms (*p* = 0.03), the time to 10% tissue activation from 2.40 ± 0.25 to 1.46 ± 0.26 ms (*p* = 0.02), and the time to 20% tissue activation from 3.91 ± 0.52 to 2.27 ± 0.33 ms (*p* = 0.02) (Figure 6C), but at all points ≥30% tissue activation, there is no significant difference in activation time (Figures 6A,B).

We also wanted to explore whether the normal conduction pathways in the tissue remained intact during optogenetic pacing. Using ElectroMap, we computed local conduction velocities using the multi-vector method (Bayly et al., 1998) with a 5 × 5-pixel grid size. The magnitude of conduction velocity vectors (Figure 6D) did not change significantly with optogenetic pacing from 26.9 ± 3.3 before to 28.6 ± 2.5 cm/s during pacing (*p* = 0.34), and there were no major changes in the directionality of conduction vectors either (Figure 6E). In summary, we report the possibility of establishing a novel left-shifted leading pacemaker site, with only minimal, controlled disturbance to normal tissue activation rate, and little or no disturbance to the pathways along which conduction normally propagates.

**Figure 7:**
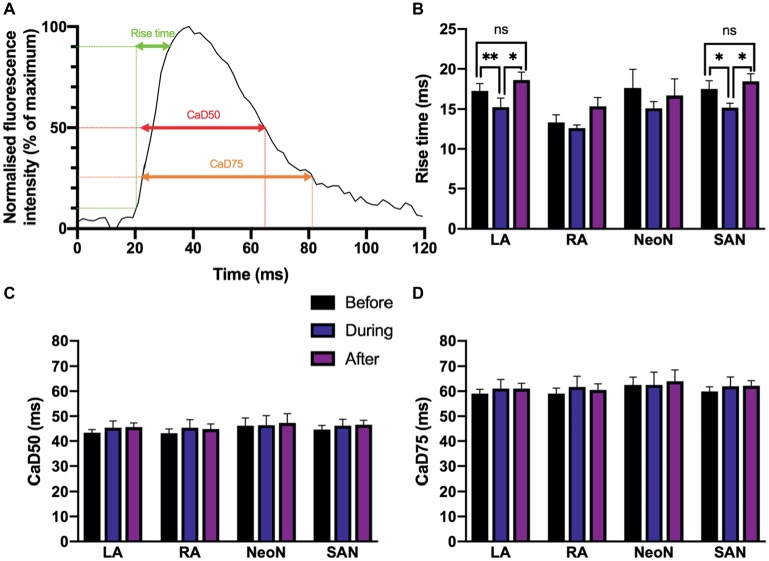
**(A)** Characteristics of Ca^2+^ transients recorded from different regions under sinus rhythm and optogenetic pacing. Representative Ca^2+^ transient illustrating rise time; CaD50 (Ca^2+^ transient duration to 50% of the decay); and CaD75 (Ca^2+^ transient duration to 75% of the decay). CaD50 and CaD75 are calculated from max. dF/dt (max. upstroke) as detailed in the protocol. **(B)** Mean rise time, before (*n* = 7), during (*n* = 7), and after (*n* = 6) optogenetic pacing. Key at center. Grouping is by region: LA, left atrium; RA, right atrium; NeoN, neonodal region (*n* = 4); SAN, nodal region. **(C)** Mean CaD50 values, no overall significant effect of optogenetic pacing. **(D)** Mean CaD75 values, no overall significant effect of optogenetic pacing.

We found small alterations to calcium cycling in our tissues, but for the most part, there was no significant change in the characteristics of calcium transient rise time, calcium transient duration to 50% of the decay (CaD50), and calcium transient duration to 75% of the decay (CaD75) with optogenetic pacing.We measured the rise time, calcium transient duration to 50% of the decay (CaD50), and calcium transient duration to 75% of the decay (CaD75) as illustrated in Figure 7A. Rise time is a measure of upstroke duration, calculated between 10 and 90% of time to peak, which minimizes analytical uncertainty (Choi and Salama, 2000; Jaimes et al., 2016). We used a mixed-effects model, with Tukey’s *post hoc* tests where required, to examine whether there were any significant effects of optogenetic pacing. For rise time, there was a significant overall effect of optogenetic pacing (*p* < 0.0001). *Post hoc* testing revealed that there was no significant effect of pacing on rise time in the right atrium or in the neonodal regions—but in the left atrium, rise time fell from 17.21 ± 0.94 ms before pacing to 15.24 ± 1.14 ms during pacing (*p* < 0.002) and rose again after pacing to 18.60 ± 1.00 ms (*p* < 0.05, during vs. after) and in the nodal region, rise time fell from 17.48 ± 1.03 to 15.24 ± 1.14 ms (*p* < 0.03) and rose again after pacing to 18.44 ± 0.95 ms (*p* = 0.02, during vs. after). In both regions, there was no significant difference between the before and after conditions (left atrium: *p* = 0.39; nodal region: *p* = 0.61)—so the reduction in rise time with optogenetic pacing is reversible upon cessation of pacing (Figure 7B). There was no overall significant effect of optogenetic pacing on either CaD50 (*p* = 0.15) or CaD75 (*p* = 0.34; Figures 7C,D). In summary, rise time is shorter in the left atrium and in the SAN region during optogenetic pacing, and this is a reversible change. Otherwise, calcium handling in the murine SAP appears to be only marginally affected.

## Discussion

The success in obtaining high quality *V*_m_ and Ca^2+^ mapping results in SAPs requires several important technical considerations, including high consumption of oxygen and high sensitivity to temperature and pH of the nodal tissue. Furthermore, the dense arrangement of nodal cells combined with the significant presence of connective tissue makes it hard for a Ca^2+^ dye to reach to the nodal and atrial cells. The critical steps include as follows:

### Tissue Preparation

Intact SAPs were micro-dissected as described previously (Ju et al., 2015). The SAN region was identified from anatomical landmarks, including the superior vena cava, the crista terminalis, and the interatrial septum. The tissue needs to be oxygenated constantly. At this point, temperature is not critical.

### Dye Loading

The Ca^2+^-sensitive dye used to load the SAP tissue was Rhod-2 AM, a dye in the acetoxymethyl ester form. Esterification of the fluorescent molecule (Rhod-2 AM) results in an uncharged molecule that can permeate cell membranes and chelate cytosolic Ca^2+^. A 100-fold increase in the molecule’s fluorescence intensity results from Ca^2+^ chelation (Choi and Salama, 2000). The voltage sensitive dye used was Di-4-ANBDQPQ, due to its favorable spectral properties for use with Rhod-2 AM. In our experiments, dye loading was carried out by incubating the tissue in a solution consisting of Pluronic F-127 dissolved in DMSO. Pluronic F-127 was incorporated into the dye loading solution to better disperse the dye into the physiological solution. Dye loading was observed to be generally better in younger mice than old, which may be related to connective tissue making up a smaller volume of the node.

The SAP’s repetitive contractions may have disrupted dye loading and/or aided the leaking of the dye out from the tissue, making it a challenge to optimize dye loading conditions. A fluorescent dye that diffuses across the cell membrane well can also exit from the cytoplasm at a high rate. Reagents like probenecid help prevent this to an extent. Combining these with optimized temperatures, timings and oxygen supply are necessary for effective loading of the tissue with dye.

### Optical Imaging

During imaging, motion artifacts caused disruption in the measurement of optical signals. Hence, to reduce contraction of the tissue, blebbistatin was used. Blebbistatin is an inhibitor of the adenosine triphosphatases associated with class II isoforms of myosin (Allingham et al., 2005). This compound has been identified as an effective excitation-contraction uncoupler, and previous investigators have used it at several different concentrations (Fedorov et al., 2007). The use of blebbistatin at 10 μM appeared to effectively abolish any motion artifact due to contraction of the SAP without harming SAP.

### Pnmt^+^ Cell-Derived Cardiomyocytes (PdCMs) and Biopacemaking Strategies

The advent of cardiac optogenetics has facilitated the identification of a new cardiomyocyte subpopulation in the murine heart, phenylethanolamine-N-methyl transferase Pnmt^+^-derived cardiomyocytes (PdCMs; Wang et al., 2017). Through selective manipulation of these cells using optogenetics, our study demonstrated how optogenetics can be used to target specific cardiomyocyte subpopulations, overcoming many of the issues associated with traditional electrophysiological techniques. Selectively stimulating PdCMs with rhythmic blue (470 nm) light pulses was sufficient to maintain sinus rhythm in murine atria, supporting previous observations from whole heart studies (Wang et al., 2017). It has also been found previously that the left atrium alone – dissected out– can be paced by light (Wang et al., 2017), but a capacity to optogenetically control the SAP has not been tested before.

The observation that a “neonode” is typically set up toward the left is consistent with previous findings that PdCMs occupy a left-sided distribution in murine heart (Wang et al., 2017). We therefore assessed the effect of pacing on left atrial activation. Optogenetic pacing of PdCMs leads to alterations in the conduction pattern across murine atria. We found (Figure 5) that optogenetic pacing of Pnmt-cre/Ai27D SAPs can often set up new sites of earliest activation (“neonodes”) toward the left of the tissue. Our proof-of-principle studies clearly demonstrate feasibility of optogenetic control of heart rate *via* the specific cell population. Indeed, in four of seven SAPs, we observed “neonode” formation, with the leading pacemaker shifting toward the left atrium. Lack of leftward shift in three of seven preparations may be due to the heterogeneity in cell numbers and clustering of the Pnmt cells in the SAPs, thus altering sink-source properties of the myocardium. While the physiological consequences of the studies presented here (specifically using Pnmt cells) are not immediately clear, application of such approaches is of interest to the community and may lead to important findings on the consequences of the spatial shift in the leading pacemaker.

As recently reviewed by Boyle et al. (2018), initial work in this area assessed possibility to override the intrinsic pacing frequency of a beating heart using optical stimuli. Bruegmann et al. (2010) explored this question in a preclinical model by generating ChR^2+^ transgenic mice. They showed in an open-chest configuration that pulses of light from a focused light source could be used to maintain ventricular activation at rates faster than intrinsic sinus rhythm. Light-induced wavefronts were initiated from different parts of the heart (e.g., atrial or ventricular sites). Remarkably, the authors found that 1:1 capture could be maintained when the illuminated area was small (0.05 mm^2^, ∼50 myocytes), suggesting that optogenetic pacing *via* a small population of cells is possible and robust. Several recent studies also provided evidence for the development of optogenetic defibrillating device for termination of tachyarrhythmias (Nyns et al., 2016; Bruegmann et al., 2017). Bruegmann et al. (2017) reported optogenetic termination of atrial tachyarrhythmia in intact hearts from transgenic as well as wild-type mice *ex* and *in vivo.* Thus, they suggested that their report could lay the foundation for the development of implantable devices for pain-free termination of AF. Nyns et al. (2016) demonstrated that forced expression of a light-gated depolarizing ion channel (ReaChR) in the adult rat heart allows contact- and shock-free termination of VTs through brief local illumination of the ventricular surface, i.e., without relying on conventional drugs, tissue ablation, or electroshocks. Both mono- and polymorphic VTs could be terminated in an effective and repetitive manner by a light-induced electrical current driven by natural electrochemical gradients, providing proof-of-concept for biological arrhythmia termination. Our study also provides proof-of-concept evidence supporting the development of light-based pacing devices targeted to a particular cellular population.

### Limitations of the Protocol

There are a number of possible limitations in this protocol including: (1) possibility of injury during the dissection of the preparation; (2) effectiveness of dye loading may vary between preparations, thereby affecting the results; and (3) partial uncoupling (reduction in conductance) due to the use of blebbistatin, which could increase the regional differences in Ca^2+^ dynamics in SAPs.

### Conclusions and Future Directions

Here, we have described a detailed protocol for dual optical mapping of murine SAPs with optogenetic pacing. Combination of high-resolution optical imaging with cell-specific optogenetic stimulation allows unique spatial control and study of electrophysiology of the SAP, not possible with traditional direct or field electrode techniques. In the present protocol, we have demonstrated this principle with PdCMs; however, the protocol can be valuable for any mouse models that cell specifically express opsins such as ChR2 within the sinoatrial region of the heart. Therefore, such an approach represents a powerful tool for enhanced electrophysiological understanding of supraventricular physiology and pathophysiology.

Future improvements may focus on exploring dual dye imaging and improved image processing software. Higher resolution and novel optical imaging modalities for 3D optical mapping are also important future directions of optical mapping.

## Ethics Statement

Pnmt-Cre mice used in this study are as described in our previous paper (Wang et al., 2017). Animals used were <6 months old, 20–25 g. All procedures including animal subjects have been approved by Institutional Animals Ethics Committees at Southwest Medical University, Luzhou, China or Department of Pharmacology at University of Oxford and the national guidelines under which the institution operates. All mice used in this study were maintained in a pathogen-free facility at Southwest Medical University or University of Oxford. Mice were given *ad libitum* access to food and water. The authors confirm that they have taken all steps to minimize the animals’ pain and suffering. Our work complies with the journal policy and regulations.

## Author Contributions

RD, RM, AR, and SH performed the experiments and produced the data. CO’S, AR, and DP analysed the results. AG-R, KD, XT, and KK assisted with data processing and manuscript preparation. ML and XO drafted the manuscript with the assistance of RM, DP, and AR. ML revised the manuscript with the assistance of CO’S, DP, RM, AR, and AG-R.

### Conflict of Interest Statement

The authors declare that the research was conducted in the absence of any commercial or financial relationships that could be construed as a potential conflict of interest.
